# Protective Immunity and Persistent Lung Sequelae in Domestic Cats after SARS-CoV-2 Infection

**DOI:** 10.3201/eid2702.203884

**Published:** 2021-02

**Authors:** Shiho Chiba, Peter J. Halfmann, Masato Hatta, Tadashi Maemura, Shufang Fan, Tammy Armbrust, Olivia M. Swartley, LaTasha K. Crawford, Yoshihiro Kawaoka

**Affiliations:** University of Wisconsin–Madison School of Veterinary Medicine, Madison, Wisconsin, USA (S. Chiba, P.J. Halfmann, M. Hatta, T. Maemura, S. Fan, T. Armbrust, O.M. Swartley, L.K. Crawford, Y. Kawaoka,);; University of Tokyo Institute of Medical Science, Tokyo, Japan (Y. Kawaoka)

**Keywords:** COVID-19, coronavirus disease, SARS-CoV-2, severe acute respiratory syndrome coronavirus 2, viruses, respiratory infections, zoonoses, cats, reinfection, protective immunity, lung sequelae

## Abstract

Severe acute respiratory syndrome coronavirus 2 readily transmits between domestic cats. We found that domestic cats that recover from an initial infection might be protected from reinfection. However, we found long-term persistence of inflammation and other lung lesions after infection, despite a lack of clinical symptoms and limited viral replication in the lungs.

Previous studies have demonstrated the transmissibility of severe acute respiratory syndrome coronavirus-2 (SARS-CoV-2) by direct or indirect contact between domestic cats (*1*,*2*). Given the close relationship between cats and humans, further characterization of the biology of SARS-CoV-2 in cats is warranted.

We inoculated domestic cats with SARS-CoV-2, and on postinfection days 3, 6, and 10, sampled organs to titrate virus (Appendix Figure 1). In plaque-forming assays in VeroE6/TMPRSS2 cells, infectious viruses were detected in the nasal turbinates and trachea of all animals on day 3, and most on day 6, whereas virus detection in the lungs was limited on day 3 and absent on day 6 (Appendix Figure 2, panel A). These results suggest that the virus replicated efficiently in upper respiratory organs, which might contribute to its high transmissibility among cats. Infectious virus was cleared from the upper and lower respiratory organs by day 10 (Appendix Figure 2, panel A). No animal showed any signs of respiratory illness during the study (Appendix Figure 3). Infectious virus was not detected (detection limit 10 pfu/g of tissue) in other examined organs (e.g., brain, liver, spleen, kidney, small and large intestine, heart, and eyelids). Viral antigen was detected in nasal turbinates and trachea but was sparse within the lungs at day 3 (Appendix Figure 4).

We conducted histopathologic examination of the lungs, trachea, and nasal turbinates. Lymphocytic inflammation within the tracheal submucosa was present on days 3 to 10, whereas lymphocytic to mixed inflammation in the nasal cavity was more severe on days 3 and 6 but minimal on day 10. In lungs, moderate lesions persisted despite clearance of virus. On day 3, we observed mild bronchitis with lymphoid hyperplasia, moderate to severe histiocytic bronchiolitis with partial to complete occlusion of lumina, and moderate to severe thickening of alveolar septa (Appendix Figure 2, panel B; Appendix Figures 4, 5). Interstitial inflammatory infiltrate decreased significantly over time (p = 0.0012, *F* = 34.70, by 1-way analysis of variance) (Appendix Figure 2, panel C); however, by day 10, alveolar septa remained thickened (Appendix Figure 5). Bronchiolitis remained with partial occlusion of bronchioles, even in regions with minimal alveolar lesions (Appendix Figure 2, panel B).

Because SARS-CoV-2 did not cause acute lethal respiratory disease in the cats in our study, cats are a compelling animal model for studying the long-term effects of nonfatal infections. Cats were infected with SARS-CoV-2 and euthanized at postinfection day 28 (Appendix Figure 6, 7). Persistent lung lesions were observed 28 days after infection, including histiocytic bronchiolitis with luminal plugs and thickened alveolar septa, similar to lesions observed on day 10 but with more chronic features such as peribronchiolar fibrosis and vascular proliferation within the thickened interstitium. We observed a notable dearth of fibrosis within alveolar septa, in contrast to what has been reported for humans with severe acute respiratory syndrome or Middle East respiratory syndrome (*3*,*4*). One cat had severe pneumonia with fibrin in alveolar spaces and endothelialitis (Appendix Figure 8), similar to what has been reported in humans with fatal coronavirus disease (*5*), although this cat did not show any respiratory signs.

To determine whether previous infection provides protection from future potential infection by SARS-CoV-2, we performed a reinfection study with 2 groups of cats. We previously reported that SARS-CoV-2 was transmitted from cats inoculated with the virus to cohoused, naive cats (*1*). In the previous study, the 3 cats that had been inoculated with SARS-CoV-2, whose nasal swabs were virus-negative on day 6 or 7 after the initial infection (*1*), were reinoculated with the same virus 4 weeks after the initial infection (Figure 1; Figure 2, panel A). No infectious virus was detected in the nasal or rectal swabs after reinfection, suggesting that the animals were protected from reinfection. These cats were euthanized at 21 days after reinfection (49 days after the initial infection), and tissue was submitted for histopathologic examination. The reinfection group showed lesions that were comparable with lung lesions observed on day 28 but with less severe thickening of alveolar septa (p = 0.041, by unpaired *t*-test) (Figure 1; Figure 2 panel B). The 3 cats in the other group, which recovered from infection that was transmitted by contact with virus-inoculated cats, were reinfected with the virus at ≈4 weeks (29–32 days) after transmission. On day 3 after reinfection, organs were harvested; infectious virus was not detected (detection limit 10 pfu/g of tissue) in respiratory organs or other organs analyzed (e.g., brain, liver, spleen, kidney, small and large intestine, heart, and eyelids). These results suggest that virus infection by natural transmission between cats, as well as by experimental inoculation, induces protective immunity against a second SARS-CoV-2 infection.

**Figure 1:**
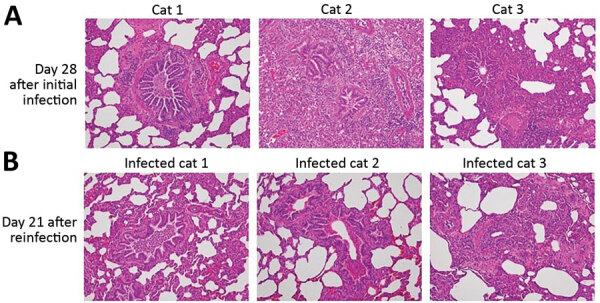
Comparison of histopathology between cats on day 28 after initial infection with severe acute respiratory syndrome coronavirus 2 and on day 21 after reinfection. Bronchioles and alveoli of cats (cats 1–3 in Appendix Figure 6) on day 28 after initial infection (A) and those of cats (infected cats 1–3 in Appendix Figure 6, upper half) on day 21 after reinfection (49 days after the initial infection) (B); original magnification × 20. Cats from both groups showed histiocytic bronchiolitis with occlusive plugs, peribronchiolar fibrosis, and thickening of alveolar septa. Mild acute hemorrhage was detected in affected and less affected regions of the lung on day 21 after reinfection, with a trend toward an increase compared with day 28 (severity score 1.8 + SEM 0.8 on day 21 vs. 0.3 + SEM 0.2 on day 28; p = 0.187 by unpaired *t*-test).

**Figure 2:**
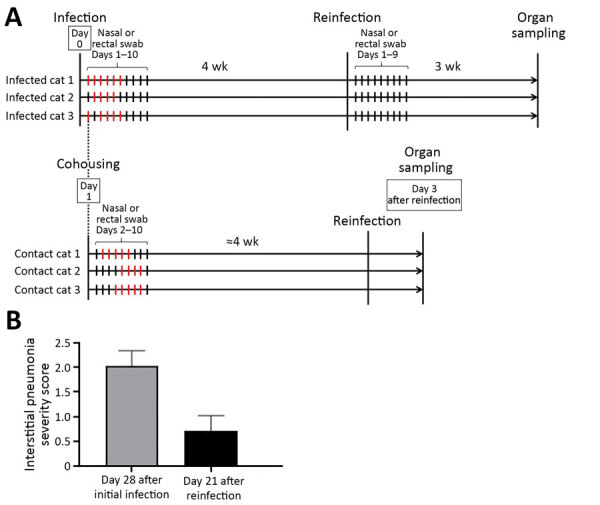
Timeline of severe acute respiratory syndrome coronavirus 2 infection and reinfection of cats and distribution of interstitial thickening. A) Timeline of infection and reinfection. As reported previously (*1*), a group of cats was inoculated with severe acute respiratory syndrome coronavirus 2 on day 0 (infected cats 1–3, upper half). A virus-naive cat was cohoused with each of the infected cats from day 1 (contact cats 1–3, lower half). The days on which infectious virus was detected in the nasal swabs are shown as red bars for each animal. In this study, we infected the cats with the same severe acute respiratory syndrome coronavirus 2 isolate at ≈4 weeks after initial infection or exposure to infected cats. After reinfection of the group shown in the upper half of the figure, no infectious virus was detected in the nasal swabs. The cats were confirmed to be seronegative before the initial infection or cohousing with infected cats, and seropositive before reinfection, on the basis of neutralization assay results. B) The distribution of interstitial thickening (interstitial pneumonia severity score) was decreased on day 21 after reinfection compared with day 28 (p = 0.041 by unpaired *t*-test).

In conclusion, SARS-CoV-2 replicated effectively in the upper respiratory tract in cats, and infectious virus was cleared from the lungs within 6 days of infection; however, histopathologic examination demonstrated chronic lung sequelae in cats even a month after viral clearance. After initial infection with SARS-CoV-2, cats were protected from reinfection, with no virus replication in respiratory organs and no additional lung damage.

Appendix Additional information about protective immunity and persistent lung sequelae in domestic cats after SARS-CoV-2 infection.
