# Supplementary material for: Protective Immunity and Persistent Lung Sequelae in Domestic Cats after SARS-CoV-2 Infection
Source: Emerg Infect Dis. 2021 Feb;27(2):660–3. doi: 10.3201/eid2702.203884 (PMC7853591; doi:10.3201/eid2702.203884)
Supplement: Appendix — Additional information about protective immunity and persistent lung sequelae in domestic cats after SARS-CoV-2 infection. [file 20-3884-Techapp-s1.pdf]

Article DOI: https://doi.org/10.3201/eid2702.203884

Protective Immunity and Persistent Lung Sequelae in Domestic Cats after SARS-CoV-2 Infection

Appendix

Materials and Methods

Virus

The SARS-CoV-2 isolate UT-NCGM02/Human/2020/Tokyo was isolated in VeroE6 and passaged twice on VeroE6 cells.

Cells

Vero E6/TMPRSS2 cells were obtained from the National Institute of Infectious Diseases, Japan. Cells were maintained in Dulbecco's modified Eagle's medium (DMEM) containing 10% fetal bovine serum (FBS) and antibiotic/antimycotic (anti/anti) solution as well as G418 (1 mg/ml).

Cats

Animal studies were approved and performed in accordance with the Animal Care and Use Committee guidelines at the University of Wisconsin-Madison. The male and female domestic cats (15–18-week-old) used in these transmission and re-infection studies were specific-pathogen-free animals from a research colony maintained at the University of Wisconsin-Madison. The male domestic cats (19-week-old) used in the virus replication and pathology study were purchased from Marshall BioResources. All animals were housed with 48%–65% humidity at 23°C, and with at least 15.2 air exchanges per hour. Weight and body temperature were recorded daily.

Experimental Infection of Cats

Under ketamine and dexdomitor anesthesia, cats were inoculated with 5.2×10^5 plaque-forming units (PFU) of SARS-CoV-2 via a combination of inoculation routes for every animal (nasal [100 µl per nare], tracheal [500 µl], oral [500 µl], and ocular [inoculation in the eyes; 50

µl per eye]). To reverse the effects of the anesthesia, antisedan was administered to the animals after completion of the inoculation. No control (mock-infected) cats were included in this study.

Swab Sample Collection

In the re-infection study, nasal and rectal swabs were collected daily after re-infection (Days 1–9). The swabs were soaked in DMEM for seconds before obtaining the nasal and rectal samples. After collection, the swabs were placed in a tube containing 1.0 ml of DMEM containing anti/anti solution and vortexed for 1 minute in preparation for the virus titration assay.

Virus Titration Assay

Confluent Vero E6/TMPRSS2 cells in 12-well plates were infected with 100 µl of undiluted or 10-fold dilutions (10^{-1} to 10^{-5}) of the homogenized organ samples or the nasal or rectal swab samples. After a 30-minute incubation, the virus inoculum was removed, the cells were washed once, and then overlaid with 1% methylcellulose solution in DMEM with 5% FBS. The plates were incubated for 3 days, and then the cells were fixed and stained with 20% methanol and crystal violet to count the plaques.

Pathological Examination

Cats were euthanized and tissues collected using standard biocontainment protocols. All cats were deeply anesthetized with the combination of ketamine and dexdomitor, and euthanized by exsanguination by bleeding from cervical artery. Macroscopic lesions were detected at all time points in lungs (data not shown). Lungs were infused with 10% formalin phosphate buffer solution via the trachea before immersion fixation in formalin along with trachea, a segment of the skull containing the nasal cavity, and other tissues. Lungs were trimmed to generate 6 representative sections of lung, including one section of the cranial and caudal segments of the left lung lobe and one from each remaining lobe. Each section included at least one grossly visible bronchus. The trachea was trimmed to generate a single cross section and the skull was trimmed to obtain a single cross section containing bilateral nasal turbinates. Standard paraffin embedded tissues were sectioned to generate slides stained with hematoxylin and eosin (HE). Selected slides were also stained with Gomori’s trichrome stain. After initial evaluation, each section of lung, trachea, and nasal cavity was scored by one of two board-certified veterinary pathologists who were both blinded to the identity of the animals and experimental groups. The distribution of rhinitis, tracheitis, and thickened alveolar septa beyond 2x normal was scored on a

scale of 1 to 5 with 1 affecting <5%, 2 affecting 5%–20%, 3 affecting 20%–50%, 50%–80%, and 5 affecting >80% of the evaluated tissue. A score of 0–5, where 0 = absent, 1 = minimal, 2 = mild, 3 = moderate, 4- marked, 5 = severe or fulminating, was given for other lesions including bronchitis, bronchiolitis with occlusive plugs, interstitial inflammatory infiltrate, and hemorrhage. Inflammation was scored based on the degree of inflammatory infiltrate; the type of inflammatory infiltrate was characterized separately, based on the predominant inflammatory cell types. Megakaryocytes were enumerated across ten 20X fields, each measuring 800 microns in diameter. For the lung, scores were averaged across lung sections for each individual, then averaged across each experimental group. The nasal turbinates and trachea were only evaluated on day 3, 6, and 10 post-infection. Statistical analysis was conducted using Prism 8 software (GraphPad Software, San Diego, CA). Corrections for multiple t-test comparisons were not used when the comparisons were complementary.

Immunofluorescent Staining

A representative tissue section from a cat in each major experimental group was selected. Sections were deparaffinized and then subject to heat-induced antigen retrieval in pH 6 citrate-buffer followed by a standard immunofluorescent staining protocol to detect SARS-CoV viral antigen in tissue sections. This included block in 5% normal donkey serum in 0.1% triton-X in phosphate buffered saline blocking solution, overnight room temperature incubation in 1:500 Rabbit polyclonal SARS Nucleocapsid protein antibody (Novus Biologicals, NB100–56576), 2 hour incubation in 1:500 Donkey anti-Rabbit Alexa 647 secondary antibody (Jackson ImmunoResearch Labs, A31573), quenching of tissue autofluorescence with TrueVIEW quenching kit (Vector Labs, SP-8400–15), and several buffer washes between each step. Epifluorescent images were acquired on Thunder 3D Tissue microscope (Leica Microsystems) using LAS X software (Leica Microsystems).

Biosafety Statement

The recombinant DNA protocol for the use of the virus was approved by the University of Wisconsin-Madison's Institutional Biosafety Committee. The cat transmission study with SARS-CoV-2 was performed in biosafety level 3 agriculture (BSL-3Ag) laboratories at the Influenza Research Institute, the University of Wisconsin-Madison. These laboratories are approved for such use by the Centers for Disease Control and Prevention. The BSL-3Ag facility

used was designed to exceed the standards outlined in *Biosafety in Microbiological and Biomedical Laboratories* (5th edition).

Features of the BSL-3Ag facility include controlled access, entry/exit through a shower change room, effluent decontamination, negative air-pressure, double-door autoclaves, gas decontamination ports, HEPA-filtered supply and double-HEPA-filtered exhaust air, double-gasketed watertight and airtight seals, and airtight dampers on all ductwork. The structure of the BSL-3Ag facility is pressure-decay tested regularly.

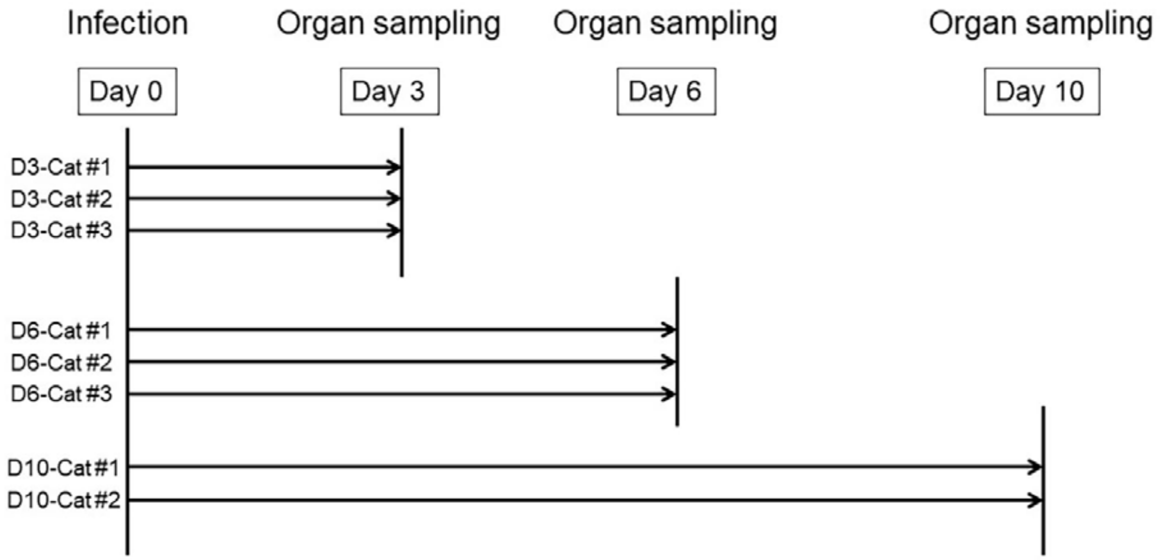

Appendix Figure 1. Timeline of cat infection and organ sampling in this study.

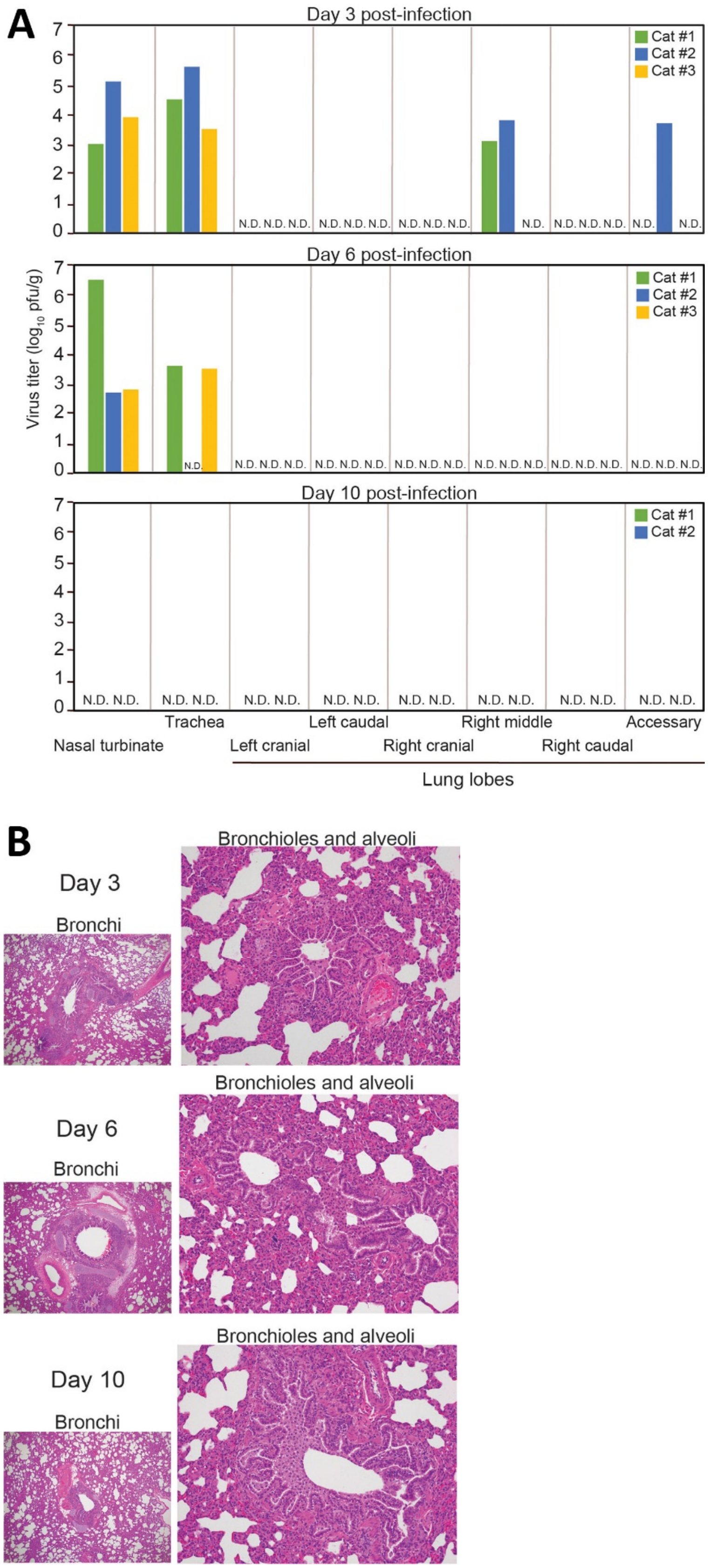
A
Day 3 post-infection
Cat #1
Cat #2
Cat #3
Day 6 post-infection
Day 10 post-infection
Virus titer (log_{10} pfu/g)
Nasal turbinate
Trachea
Left cranial
Left caudal
Right cranial
Right middle
Right caudal
Accessary
Lung lobes
N.D.
B
Day 3
Bronchi
Bronchioles and alveoli
Day 6
Bronchi
Bronchioles and alveoli
Day 10
Bronchi
Bronchioles and alveoli

C

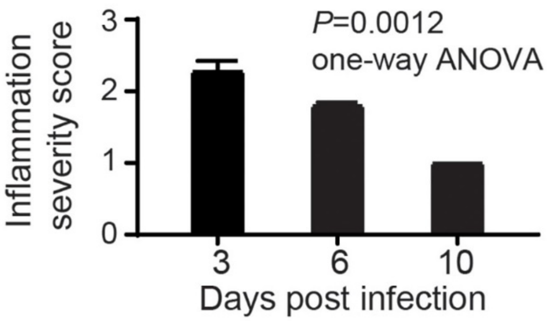

Appendix Figure 2. Replication and pathogenicity of SARS-CoV-2 in cat respiratory organs. (A) Cats were inoculated with SARS-CoV-2. On Days 3, 6, and 10 post-infection, their organs were collected to assess infectious virus titers. N.D., infectious virus not detected (<10 pfu/g). (B) Typical images of hematoxylin and eosin (HE)-stained bronchi, bronchioles, and alveoli on days 3, 6, and 10 post-infection. Bronchitis was mild with lymphoid hyperplasia that diminished over time and sloughed epithelial cells in the lumen at day 3 (see Appendix Figure 4) were minimal to absent at day 6 and day 10. Bronchiolitis was persistent throughout all examined time points with partial and, rarely, complete occlusion of bronchiolar lumens by sheets of epithelioid-like macrophages that were occasionally continuous with macrophages in the bronchiole walls and peribronchiolar connective tissue. Thickening of alveolar septa was likewise persistent (see Appendix Figure 5). (C) Interstitial inflammatory infiltrate significantly decreased in lungs over time, as indicated by the inflammation severity score.

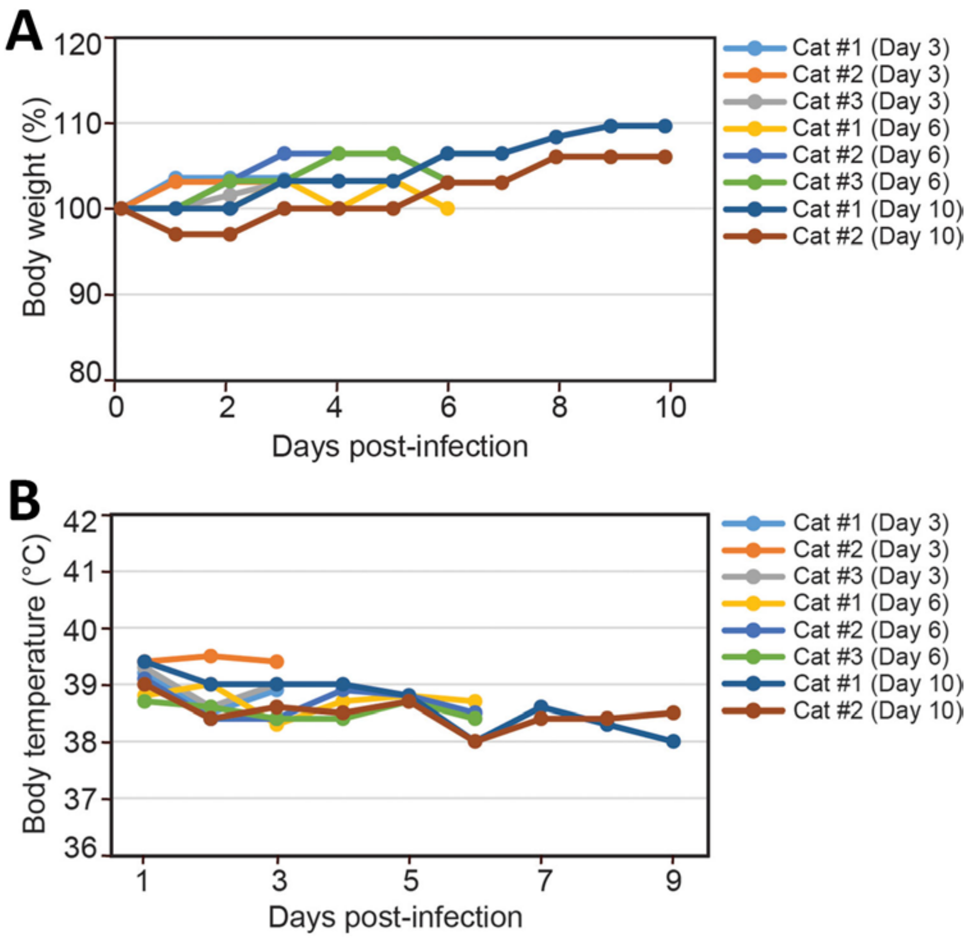

Appendix Figure 3**.** Changes in bodyweight (A) and body temperature (B) of SARS-CoV-2-infected cats euthanized on day 3, 6, or 10 post-infection.

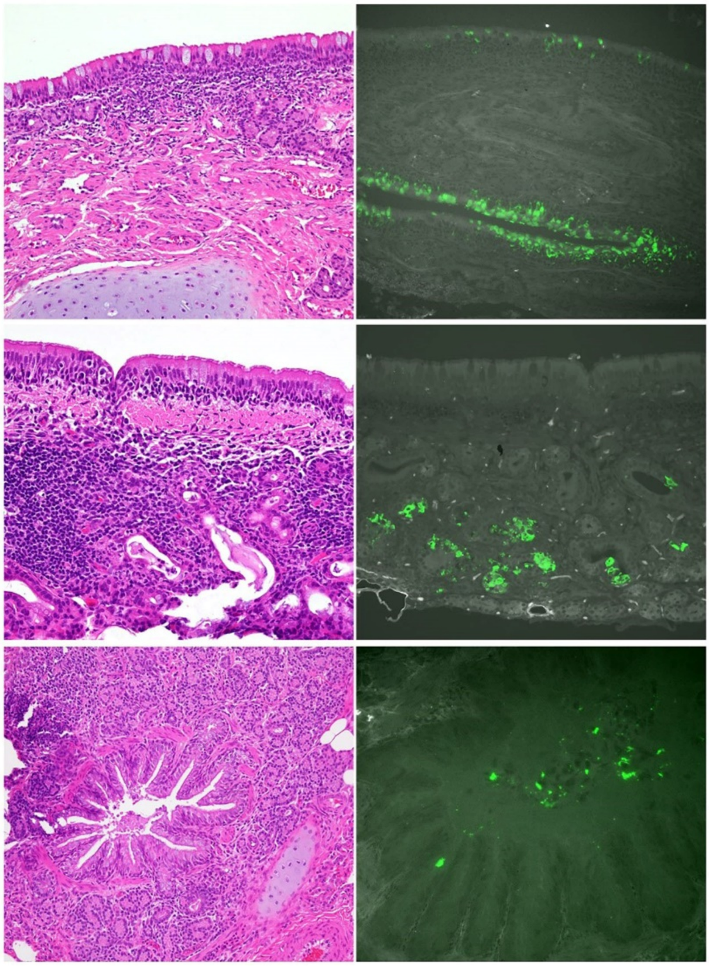

Appendix Figure 4**.** At day 3, viral antigen was detected in regions corresponding to lesions of the nasal turbinates and trachea but was not detected in lesions in the lung. In the nasal cavity, inflammation was in the superficial submucosa and frequently infiltrated the epithelium (top panel, left). Viral antigen was detected in the nasal epithelium, within cells suspected to be olfactory epithelial cells (top panel, right). In the trachea, inflammation was deeper within the submucosa surrounding and occasionally effacing submucosal glands (middle panel, left). Viral antigen in the trachea was detected within submucosal glandular epithelium (middle panel, right). Similar distribution and character of inflammation was present in the trachea at day 6 and day 10, while inflammation trended toward a decrease to minimal by day 10 in the nasal turbinates (data not shown). In the lung, bronchitis consisted of mild inflammation within submucosal glands along with lymphoid hyperplasia (lower, left). Viral antigen was sparse in the lung and was only found in sloughed epithelial cells and debris in the lumen of bronchi and scattered along the apical surface of the bronchial epithelium (lower panel, right). Viral antigen was not detected in the bronchioles or alveoli. HE images on the left, 20X magnification. On the right, immunofluorescent staining for viral antigen was pseudocolored green during image acquisition with a far red light source and merged with a greyscale image of autofluorescence acquired with GFP light source; nasal and trachea 20X magnification, bronchus 40X magnification.

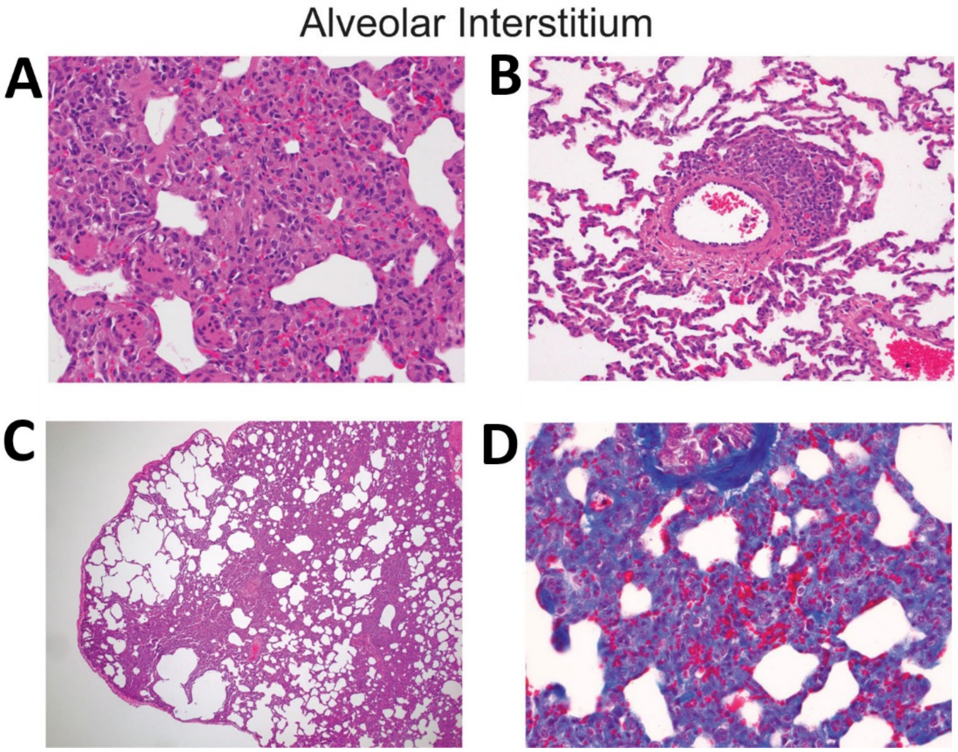

Appendix Figure 5. The alveolar interstitium is thickened after SARS-CoV-2 infection. (A) At day 3, in patchy to coalescing regions of the lung, the alveolar septa are thickened by a variable combination of type II pneumocytes, abundant macrophages, moderate numbers of neutrophils, and fewer lymphocytes, along with stromal cells that are suspected to be vascular precursors. Respiratory bronchioles were often lined by attenuated epithelium as seen in the lower left aspect of the image. Intravascular megakaryocytes (not shown) decreased slightly over time (ranging from 8.0 to 3.4 per ten 20X fields, $p = 0.189$ one way ANOVA). HE, 40X magnification. (B) Multifocal perivascular lymphocytic nodules admixed with scattered macrophages were present, sometimes in lesser affected areas of the section. Perivascular lymphocytic nodules in more severely affected regions occasionally extend into and thicken adjacent alveolar septa (not shown). Vascular endothelium was diffusely reactive at all timepoints examined, often ranging from plump to vacuolated. HE, 20X magnification. Images in (A) and (B) are from day 3, Cat #3. (C) By day 10, distention of alveolar spaces was seen in some regions adjacent to patchy atelectasis while other regions of the lung showed thickened alveolar septa with a notable lack of fibrosis. HE 4X magnification. (D) On trichrome stain, mature collagen and smooth muscle stain dark blue, while dark blue is notably sparse to absent within the thickened interstitium, confirming that fibrosis is minimal. Gomori trichrome stain, 40X magnification. Images in (C) and (D) are from day 10, Cat #1.

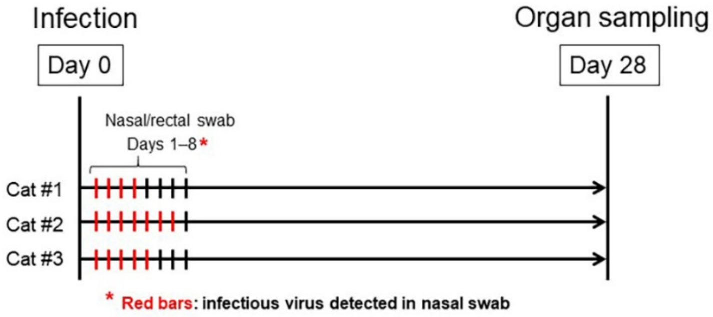

Appendix Figure 6**.** Timeline of cat infection and organ sampling in this study. On days 1–8 post-infection, nasal and rectal swabs were taken from the cats and infectious virus was titrated by performing plaque assays. The days on which infectious virus was detected in the nasal swabs are shown as red bars for each animal. The animals were confirmed to be seronegative by performing a neutralization assay before they were infected.

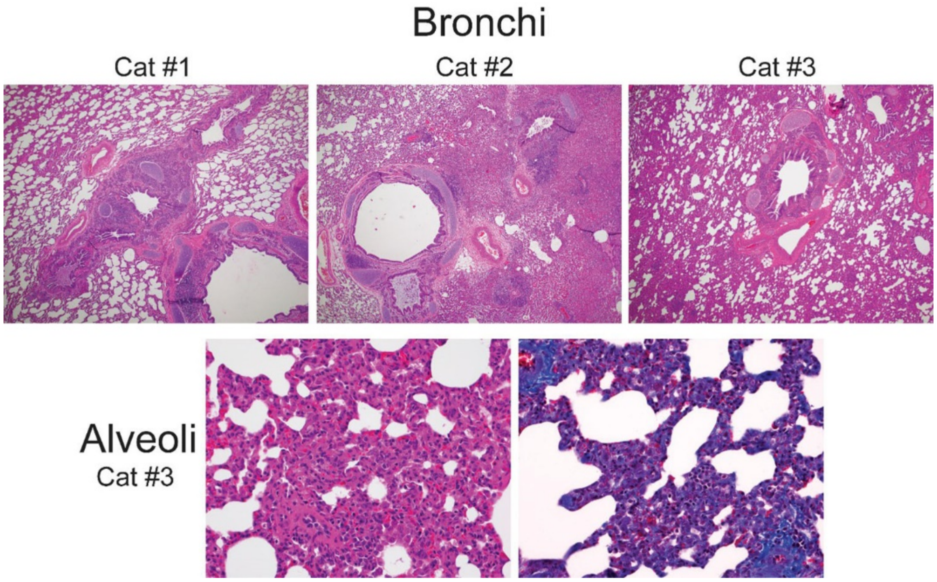

Appendix Figure 7**.** Histopathology of the lungs of cats at 28 days post-infection (Cats #1–3 in Appendix Figure 6). Histopathology was consistent with that seen at 10 days post-infection with more chronicity, including multifocal histiocytic bronchiolitis with luminal plugs and regional thickening alveolar septa (top panel, 4X magnification). Alveolar septa were thickened by macrophages, lymphocytes, and fewer neutrophils and plasma cells with increased stromal cells suggestive of vascular proliferation (lower panel, 40X magnification). Gomori trichrome stain, wherein mature collagen and smooth muscle stains dark blue, demonstrates mature collagen surrounding medium caliber vessels, foci of smooth muscle within the tips of alveolar septa and in respiratory bronchioles, and a notable lack of fibrosis within the thickened interstitium (lower panel, right, 40X magnification). Lung lesions were particularly severe in Cat #2 (see Appendix Figure 8).

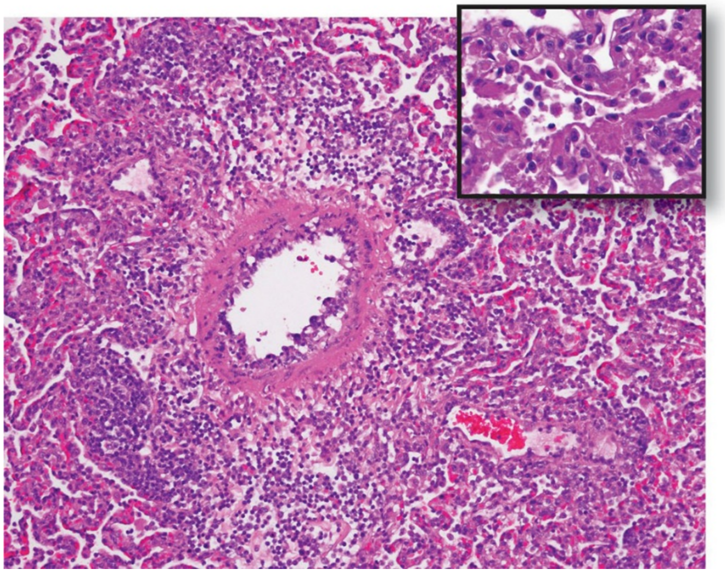

Appendix Figure 8**.** The lung lesions of one cat at day 28 was particularly severe. Lung pathology in a single cat (Cat #2 in Appendix Figures 6 and 7) was particularly severe with endothelialitis (vasculitis), severe perivascular inflammation and edema, and alveolar collapse with macrophages, lymphocytes, plasma cells and lower numbers of neutrophils within alveolar spaces. HE, 20X magnification. Inset shows PAS stain of accumulation of fibrin and cellular debris along with inflammatory cells within alveolar spaces. PAS stain, 40X magnification.
